# Short-Term Feeding Disruption Effects and Efficacy of Six Biopesticides Against *Empoasca onukii* (Hemiptera: Cicadellidae)

**DOI:** 10.3390/biology15050419

**Published:** 2026-03-04

**Authors:** Zhifei Jia, Chunling Yang, Yilan Liu, Yilin Yang, Rui Zhou, Zhenzhen Cheng, Shubao Geng, Yongyu Xu, Zhenzhen Chen, Li Qiao

**Affiliations:** 1 College of Agriculture, Xinyang Agriculture and Forestry University, New 24th Street, Xinyang 464000, China; jiazf0525@163.com (Z.J.); 2023180001@xyafu.edu.cn (C.Y.); 17837577912@163.com (Y.L.); 18638374401@163.com (Y.Y.); 15836286354@163.com (R.Z.); 18336859975@163.com (Z.C.); 2018180002@xyafu.edu.cn (S.G.); 2 College of Plant Protection, Shandong Agricultural University, 61 Daizong Street, Tai’an 271000, China; xuyy@sdau.edu.cn (Y.X.); chenzz0327@163.com (Z.C.)

**Keywords:** *Empoasca onukii*, tea plants, electrical penetration graph, biological control, control efficacy

## Abstract

*Empoasca onukii* severely damages tea plants as a major sap-sucking pest, leading to the increasing adoption of biopesticides as a sustainable alternative to chemical control. However, existing research has largely focused on the final lethal effects of these agents, while their short-term interference patterns on pest feeding behavior remain unclear. In this study, six biopesticides—azadirachtin, matrine, *Beauveria bassiana*, *Metarhizium anisopliae* CQMa421, *Mamestra brassicae* nucleopolyhedrovirus (MbNPV), and *Bacillus thuringiensis* (Bt)—were evaluated using the electrical penetration graph (EPG) technique to precisely analyze their interference on the short-term (6 h) feeding behavior of *E. onukii*, alongside field trials to validate control efficacy. EPG analysis revealed that different types of biopesticides significantly disrupted feeding in distinct ways. The two botanical pesticides and CQMa421 mainly prolonged the non-probing phase (waveform Np) and reduced active non-phloem feeding (C waveform) (*p* < 0.05); Bt and *B. bassiana* significantly extended the resting phase (waveform R) and decreased the frequency of passive phloem feeding (waveform E) (*p* < 0.05), whereas MbNPV exhibited a combined effect, simultaneously prolonging both Np and R waveforms while reducing waveform C (*p* < 0.05). Field trials showed that all tested treatments achieved complete control (100%) at 21 days post-application. Moreover, across a wide range of concentrations, they all demonstrated excellent and stable control performance. These findings provide diverse agent options for the green control of *E. onukii* in tea plantations and lay a foundation for constructing a green integrated pest management system centered on biological control for tea plant pests.

## Simple Summary

The tea leafhopper is a major pest that damages tea plants by sucking sap, prompting the use of biopesticides as an eco-friendly alternative to chemical pesticides. However, past studies focused mostly on how these agents kill pests, leaving their short-term effects on feeding behavior unclear. This study tested six biopesticides, using a precise method to monitor feeding over 6 h, and conducted field trials to evaluate their effectiveness. Results showed that different biopesticides disrupted feeding in distinct ways: plant-based and fungal-based biopesticides spent increased time not feeding and reduced active feeding. Bacteria-based pesticides extended resting periods and decreased passive feeding, while a virus combined both effects. Field trials confirmed that all treatments fully controlled the pest within 21 days, performing consistently across various concentrations. These findings offer multiple green options for managing leafhoppers in tea plantations, supporting the development of sustainable pest control systems that benefit the environment and tea production.

## 1. Introduction

Tea plant [*Camellia sinensis* (L.) O. Kuntze] is a perennial plantation crop that requires a warm and humid climate for optimal growth and production. Such climatic conditions also favor pests and diseases that infest tea plants [1,2]. *Empoasca onukii* Matsuda is recognized as one of the four most damaging piercing-sucking pests, alongside *Toxoptera aurantii*, *Apolygus lucorum*, and *Aleurocanthus spiniferus*, among the major insect pests affecting tea plantations worldwide, and it occurs in tea-producing countries across Asia such as China, Vietnam, Japan, and Indonesia [3,4,5]. Female adults lay eggs by inserting their ovipositors into the tender shoots of tea plants, impeding the transport of nutrients. Both nymphs and adults cause damage by sucking sap from young buds, leaves, and stems. Lightly infested tea plants show chlorosis and premature aging of buds and leaves, severely affecting both yield and quality [6,7,8]. Currently, controlling *E. onukii* in production relies primarily on chemical pesticides such as bifenthrin, chlorfenapyr, and thiamethoxam, which readily lead to the development of pesticide resistance in *E. onukii* and even raise concerns over pesticide residues [9,10]. Non-chemical and sustainable pest management strategies are increasingly viewed as transformative for plant protection in the tea industry [10,11]. They have been incorporated into integrated pest management (IPM) programs for tea pests, including entomopathogenic bacteria, fungi, nematodes, and certain viruses, which are highly efficient, target-specific, and safe for soil, plants, and the environment [12,13,14,15]. Furthermore, pest management using biological control agents can also promote the proliferation of beneficial microbial communities in the soil, reduce soil-borne pathogens, and protect microbial diversity [16].

In addition to synthetic chemical pesticides, a wide range of biologically based alternatives have been developed and registered for pest management in tea plantations. Among these, botanical pesticides represent a major category of commercial biopesticides. Neem-based products are representative examples, and other active substances such as nicotine, rotenone, matrine, azadirachtin, veratrine, toosendanin, veratridine, limonin, eucalyptol, and citronella oil are all registered for use [10]. Entomopathogens are promising biocontrol agents against tea pests [10]. *Bacillus thuringiensis* (Bt), *Metarhizium anisopliae*, and *Beauveria bassiana* have shown significant efficacy in controlling tea pests [17]. Bt is the predominant bacterial species isolated from the cadavers of tea pests and the tea garden ecosystem. Various Bt strains have been applied in China, Japan, India, Sri Lanka, Bangladesh, and Vietnam to control lepidopteran pests in tea plantations [18]. Nucleopolyhedroviruses (NPVs) have reached commercial scale as biocontrol agents in tea plantations due to their safety and high efficacy. For instance, a *Biston suppressaria* NPV variant can infect the geometrid pests *Hyposidra infixaria* and *Hyposidra talaca*, which are newly recorded in India [19]. The *Ectropis obliqua* nucleopolyhedrovirus QF4 strain isolated in China effectively infects *E. obliqua* and *Ectropis grisescens* [20]. The *Mamestra brassicae* nucleopolyhedrovirus developed in China demonstrates good control efficacy against various lepidopteran pests, including *E. grisescens* and *Spodoptera frugiperda* [21,22,23]. *M. anisopliae* sensu lato (s. l.) has shown potential efficacy against a wide range of insects on various crops, including tea [1,24]. For example, under field conditions, a formulation of *M. anisopliae* s. l. demonstrated high activity against several pests on tea plants in Kenya [25]. The *M. anisopliae* strain CQMa421, developed in China, can infect over 80 species (or types) of agricultural, forestry, and public health pests across seven orders, including Lepidoptera, Coleoptera, Orthoptera, Hemiptera, Diptera, Hymenoptera, and Thysanoptera, at various developmental stages [26].

Biopesticides often function by interfering with and inhibiting insect feeding behavior, a critical lethal or sublethal mechanism of action [27,28]. Specifically, they can directly reduce nutrient intake and lead to starvation by suppressing feeding, damaging feeding-related physiological structures, or blocking the neuromuscular functions required for feeding. This effect synergizes with their primary toxicological actions, thereby increasing insect mortality, reducing crop damage, and potentially slowing the development of pest resistance [29,30]. It has been reported that azadirachtin acts as an antifeedant against tea pests such as *Helopeltis theivora*, *Scirtothrips dorsalis*, and *Empoasca flavescens*, while matrine functions primarily as a toxicant [31,32,33,34,35]. The core mechanism of Bt is stomach action [36,37]. In contrast, *M. anisopliae* and *B. bassiana* infect pests through contact, a mode of action which requires successful penetration of the cuticle and suppression of the host’s immune system [38,39]. A unique additional mechanism of *B. bassiana* involves secreting antimicrobial peptides that specifically inhibit Gram-positive bacteria on the insect’s body surface. This reduces interference from beneficial surface microbiota during fungal infection and significantly enhances infection efficiency [40]. However, few studies have reported the effects of infection by the aforementioned biopesticides on short-term (6–8 h) pest feeding behavior. Investigating short-term behavioral responses holds significant scientific value for the development and evaluation of biopesticides. Compared to traditional toxicity tests that rely on mortality rates, monitoring short-term behavior can reveal the sublethal effects and modes of action of pesticides earlier and more sensitively, while the observations are also directly relevant to field control efficacy [41]. The EPG technique, owing to its high temporal resolution and capacity to discriminate specific probing phases, constitutes an ideal tool for observing short-term feeding behavior [7]. By linking early behavioral disruption quantified via EPG with subsequent field efficacy data, a direct bridge can be established between laboratory-based mechanistic insights and practical pest management outcomes under field conditions.

This study utilized the electrical penetration graph (EPG) technique to analyze differences in the feeding behavior of *E. onukii* after treatment with different biopesticides (two entomopathogenic fungi, one virus, one bacterium, and two botanical pesticides) and integrated these behavioral findings with field efficacy trials to comprehensively evaluate control effects. It aims to investigate the early effects of biopesticides on pest feeding behavior, thereby providing a theoretical basis for the targeted design and optimization of pesticides.

## 2. Materials and Methods

### 2.1. Test Insects

The tea green leafhoppers and tea plants used in this study were collected from a tea plantation in Xiajiachong Village, Shihegang Town, Xinyang, China (32°18′ N, 114°35′ E). The insects were maintained in rearing cages (60 × 60 × 60 cm) within a controlled-environment insectary (24 ± 2 °C, 70–80% relative humidity, 16:8 h light:dark cycle) and fed with fresh tea shoots. Adults 5–7 days post-emergence were selected as experimental subjects. Preliminary analyses indicated no consistent sex-based differences in feeding behavior under the tested conditions. Therefore, test insects were used in random sex combinations. Each individual was used only once. The insects used in the EPG experiments and field trials were completely separate populations. No individuals were reused across the two types of experiments.

### 2.2. Test Biopesticides

The biopesticides used in this study are as follows: 0.3% azadirachtin EC (Chengdu Green Gold Biotechnology Co., Ltd., Chengdu, China), 2.0% matrine OD (Hebei Ruibaode Biochemical Co., Ltd., Shijiazhuang, China), 300 billion spores/g *Beauveria bassiana* WP (Shanxi Lühai Pesticide Technology Co., Ltd., Yuncheng, China), 80 billion spores/mL *Metarhizium anisopliae* CQMa421 OD (Chongqing Julixin Bioengineering Co., Ltd., Chongqing, China), *Mamestra brassicae* nucleopolyhedrovirus (MbNPV, Jiangxi Xinlong Biotechnology Co., Ltd., Yichun, China), and *Bacillus thuringiensis* (Shandong Yitai Biotechnology Co., Ltd., Tai’an, China).

### 2.3. EPG Test

To investigate the feeding behavior of adult *E. onukii* on tea plants treated with six different biopesticides, the following treatments were established: (1) Bt was diluted 250-fold in distilled water; (2) MbNPV was diluted 900-fold in distilled water; (3) CQMa421 was diluted 850-fold in distilled water; (4) *B. bassiana* was diluted 500-fold in distilled water; (5) azadirachtin was diluted 400-fold in distilled water; (6) matrine was diluted 125-fold in distilled water. Distilled water served as the blank control. The concentration of each biopesticide was chosen based on the manufacturer’s recommended dosage for field application against piercing-sucking pests in tea plantations. Healthy, uniformly growing tea shoots approximately 30 cm in length were selected, immersed in the prepared pesticide solutions for 30 s, and then inserted into nutrient soil in small pots. The treated plants were placed in a cool, ventilated area for approximately 5–10 min to air-dry. Eighteen plants were used for recording per treatment. Each tea shoot was infested with a single *E. onukii* adult, constituting one biological replicate. A total of 18 replicates were measured for each treatment. Tea plants of uniform growth stage were randomly assigned to different treatment groups to minimize plant variability.

Prior to the experiment, the EPG equipment was zeroed and preheated for 15 min. Test insects were starved for 1 h before the assay. A gold wire (20 mm in length, 18.5 μm in diameter; EPG Systems, Wageningen, The Netherlands) was attached to the pronotum of a leafhopper using a hand-mixed, water-based silver conductive paint glue (EPG Systems). The other end of the gold wire was connected to the top of a copper electrode (20 mm in length). The copper electrode with the attached insect was inserted into a foam board, and the insect was observed for 2–3 min to ensure secure attachment and good condition. The prepared insect was then placed on the abaxial surface of a fresh tea leaf with sufficient space for movement. The copper electrode was inserted into the input of the EPG head-stage amplifier. Another copper electrode (100 mm in length, 2 mm in diameter) was inserted into the soil of the plant container to complete the circuit. The Faraday cage was closed, and the recording was initiated using the EPG Stylet+ 01.20 software to monitor the probing behavior of *E. onukii* on the tea plant for a 6 h period.

A Giga-8 DC-EPG device (EPG Systems, Wageningen, The Netherlands) was used to monitor the probing and feeding activities of adult *E. onukii* on tea plants. Signals were digitized using a USB analog/digital converter card (DI-158U; DATAQ Instruments, Akron, OH, USA) and transmitted to a laptop computer for recording.

### 2.4. Field Trial

A field trial was conducted from 8 July to 29 July 2025 in Xiajiachong Village, Shihegang Town, Xinyang, China (32°18′ N, 114°35′ E). The tea plantation was in the peak occurrence period of *E. onukii*, received no prior pesticide applications, and the tea plants were in good growth condition. The tested biopesticides were diluted to their respective concentrations (Table 1), with distilled water serving as the control. The intermediate concentration for each biopesticide was chosen based on the manufacturer’s recommended dosage for field application against piercing-sucking pests in tea plantations. To evaluate the robustness and flexibility of each biopesticide under practical conditions, we additionally tested one lower and one higher concentration relative to the recommended rate. A completely randomized block design was employed, in which each treatment had three replicates, resulting in a total of 57 plots, each measuring 60 m^2^ and randomly allocated across treatments. To prevent interference between different treatments, plots for different agents were separated by a row of tea bushes approximately 5 m wide. Applications were performed using a motorized knapsack sprayer (Shijiazhuang Funong Machinery Co., Ltd., Shijiazhuang, China). During spraying, emphasis was placed on achieving uniform coverage on the tea canopy surface and the abaxial sides of the tender shoots. The application was conducted under suitable weather conditions, without precipitation or excessive wind. The pre-treatment pest density was determined by random sampling within each treatment area before 9:00 a.m. on the day of application. For each plot, five points were randomly selected. Using the sweep net sampling method, we employed a standard sweep net (38 cm in diameter) with a sturdy handle and a fine mesh collection bag to conduct a uniform sweep within a 5 m^2^ area at each point to count the number of *E. onukii*. Post-treatment pest populations were surveyed using the same method before 9:00 a.m. on days 3, 5, 9, 14, and 21 after treatment.

The control efficacy and population reduction rate were calculated using the following formulas:

Population Reduction Rate (%) = [(Pre-treatment Count − Post-treatment Count)/Pre-treatment Count] × 100%(1)

Control efficacy (%) = [(Reduction Rate in Treatment − Reduction Rate in Control)/(100 − Reduction Rate in Control)] × 100%(2)

### 2.5. Data Analysis

All data were analyzed in GraphPad Prism 9 software (GraphPad Software, San Diego, CA, USA). All data were tested for normality (Shapiro–Wilk test) and homogeneity of variance (Levene’s test) prior to analysis. Data on EPG were analyzed by one-way analysis of variance using Tukey’s HSD post hoc test. The field trial data were analyzed using two-way analysis of variance with Tukey’s HSD.

## 3. Results

### 3.1. Effects of Six Biopesticides on the Frequency and Duration of EPG Waveforms in E. onukii

**Figure 1:**
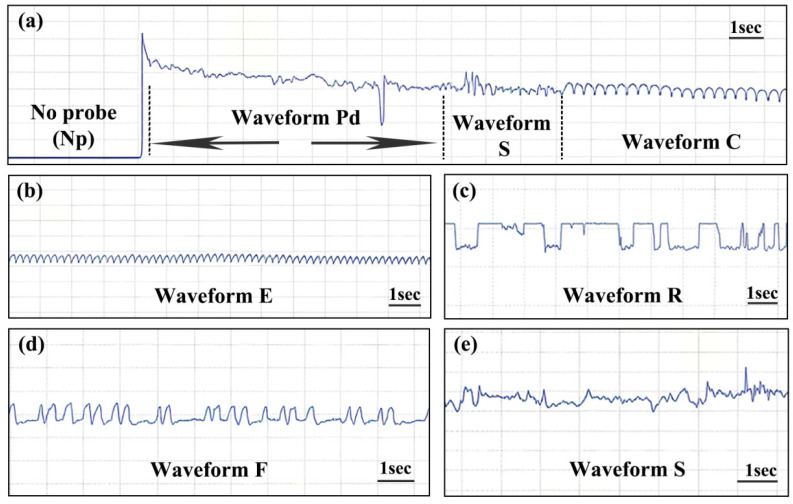
EPG recordings of *E. onukii* on tea plants. (**a**) Waveform Np (non-probing): *E. onukii* is in a non-feeding state, with no stylet probing or ingestion of plant sap. Waveform Pd (probing): The waveform preceding the initiation of feeding. Waveform C (active feeding): A waveform produced when the insect’s stylets have not fully penetrated the plant phloem but are actively ingesting sap. (**b**) Waveform E (passive feeding): This waveform indicates that the insect’s stylets have entered the plant phloem, representing a sustained but passive ingestion state. (**c**) Waveform R (resting): The insect’s stylets are not in contact with the leaf tissue or phloem, and no form of feeding is occurring. (**d**) Waveform F (mechanical obstruction): A waveform generated when the insect encounters resistance or difficulty during cell penetration. (**e**) Waveform S (salivary secretion): The insect’s stylets have penetrated the plant epidermis and are secreting saliva to dissolve plant tissues.

**Figure 2:**
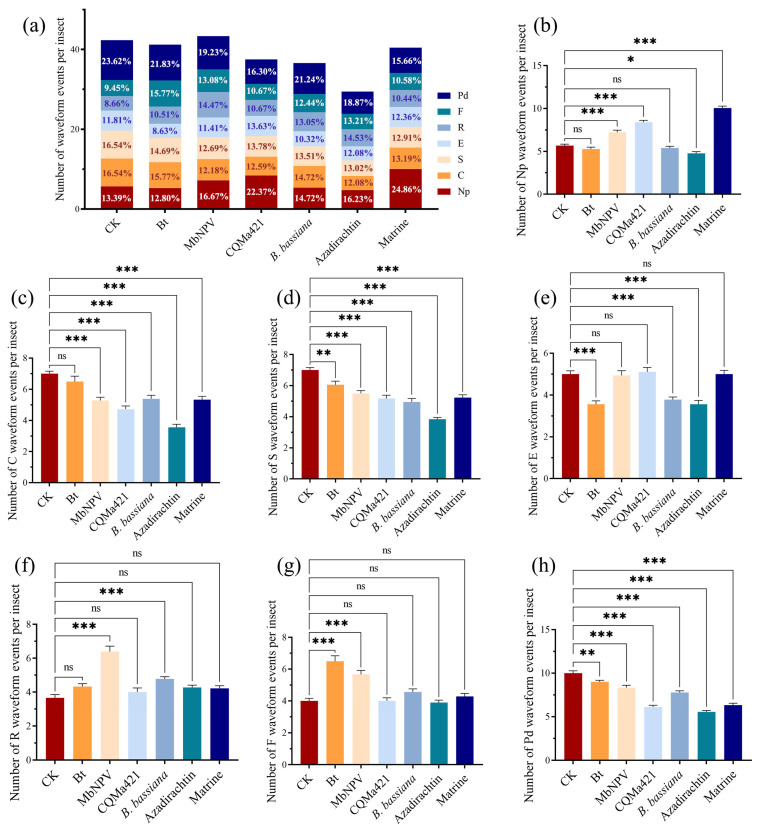
Number of probing events by adult *E. onukii* on tea plants during the 6 h recording period. (**a**) The proportion of different probing waveforms produced by adult *E. onukii* on tea plants relative to the total number of events after treatment with the six biopesticides. Number of (**b**) waveform Np, (**c**) waveform C, (**d**) waveform S, (**e**) waveform E, (**f**) waveform R, (**g**) waveform F, and (**h**) waveform Pd produced by adult *E. onukii* on tea plants after treatment with the six biopesticides. Bars indicate standard errors. All experiments were done with 18 replicates using one-way analysis of variance. *, **, and *** indicate significant differences at *p* < 0.05, *p* < 0.01, and *p* < 0.001, respectively; ns indicates a non-significant difference.

**Figure 3:**
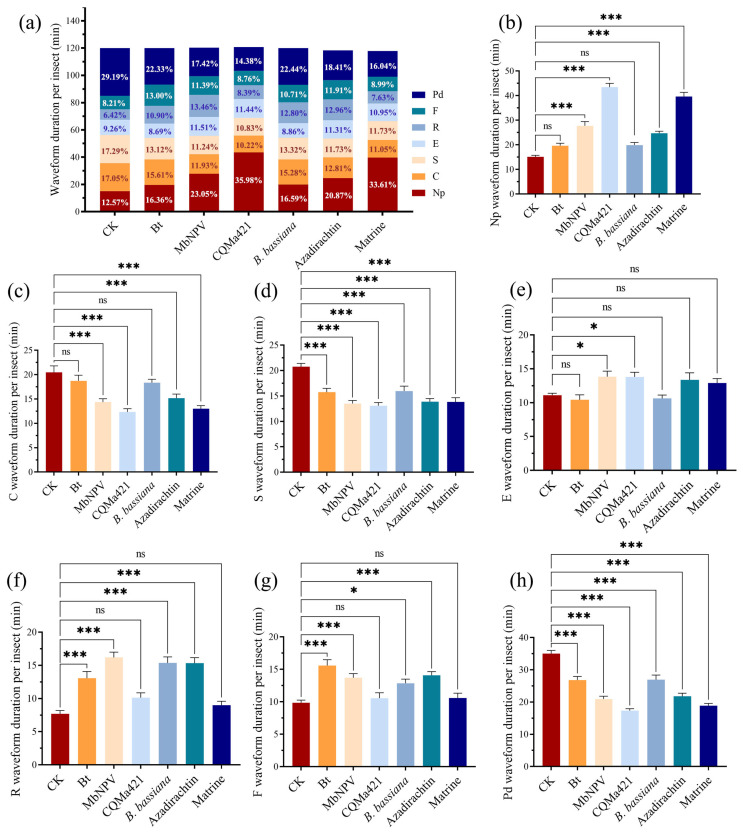
Probing duration of adult *E. onukii* on tea plants during the 6 h recording period. (**a**) Proportion of the total probing time occupied by different waveforms produced by adult *E. onukii* on tea plants after treatment with the six biopesticides. Duration of (**b**) waveform Np, (**c**) waveform C, (**d**) waveform S, (**e**) waveform E, (**f**) waveform R, (**g**) waveform F, and (**h**) waveform Pd produced by adult *E. onukii* on tea plants after treatment with the six biopesticides. Bars indicate standard errors. All experiments were conducted with 18 replicates using one-way analysis of variance. * and *** indicate significant differences at *p* < 0.05, and *p* < 0.001, respectively; ns indicates a non-significant difference.

The feeding of adult *E. onukii* on tea plants produced seven distinct waveforms: Np, Pd, C, E, R, F, and S (Figure 1). The count and total duration of waveform Np were significantly higher following treatment with MbNPV, CQMa421, and matrine compared to the control. Azadirachtin increased the total duration of waveform Np but decreased its frequency (Figure 2b and Figure 3b). All six biopesticides significantly reduced the count of S and Pd waveforms (Figure 2a,d,h) and significantly shortened their total durations (Figure 3a,d,h). With the exception of Bt, the other five biopesticides significantly reduced the number of waveform C (Figure 2c). However, among these five, only four (MbNPV, CQMa421, azadirachtin, and matrine) significantly shortened the total duration of waveform C (Figure 3c). Treatments with Bt, azadirachtin, and *B. bassiana* significantly reduced the number of waveform E (Figure 2e), while MbNPV and CQMa421 significantly increased the total duration of waveform E (Figure 3e). Bt, MbNPV, *B. bassiana*, and azadirachtin significantly prolonged the total durations of R and F waveforms (Figure 3f,g). Only Bt and MbNPV significantly increased the count of waveform F (Figure 2g), and only MbNPV and *B. bassiana* significantly increased the count of waveform R (Figure 2f).

### 3.2. Field Trial of Bt at Different Concentrations Against E. onukii

**Figure 4:**
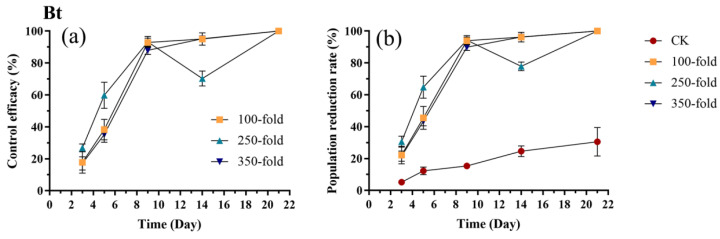
Control efficacy (**a**) and population reduction rate (**b**) of *E. onukii* following application of Bt at different concentrations (100-, 250-, and 350-fold). Data represent mean ± SE (n = 3). Bars indicate standard errors. All experiments were done in three replicates, using two-way analysis of variance.

The control efficacy of Bt against *E. onukii* increased to approximately 90% by day 9 and peaked at 100% by day 21 for all three dilutions (Figure 4a). No significant differences in efficacy were observed among the different concentrations on days 3, 9, and 21 (*p* > 0.05). On day 5, the efficacy of the 250-fold dilution was significantly higher than that of both the 100-fold (*p* = 0.003, t = 3.614) and the 350-fold dilution (*p* < 0.001, t = 4.134), reaching 59.78% (Figure 4a, Table S1). Conversely, on day 14, the efficacy of the 250-fold dilution was significantly lower than that of the 100-fold (*p* < 0.001, t = 4.154) and the 350-fold dilution (*p* < 0.001, t = 4.186), measuring 70.27% (Figure 4a, Table S1). The population reduction rates of *E. onukii* in all Bt-treated groups were significantly higher than in the control (*p* < 0.05) (Table S1). By day 9, the population reduction rates for all three concentrations reached approximately 90% (Figure 4b). However, on day 14, the reduction rate for the 250-fold dilution decreased to 77.88%. By day 21, the population reduction rates for all three concentrations reached 100% (Figure 4b).

### 3.3. Field Trial of MbNPV at Different Concentrations Against E. onukii

**Figure 5:**
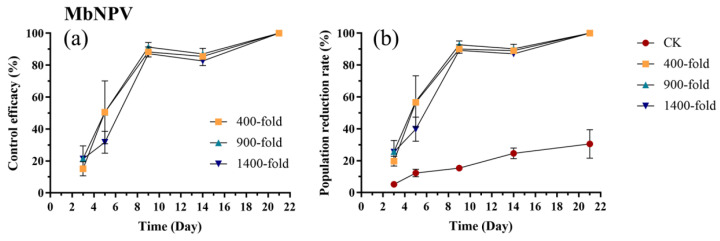
Control efficacy (**a**) and population reduction rate (**b**) of *E. onukii* following application of MbNPV at different concentrations (400-, 900-, and 1400-fold). Data represent mean ± SE (n = 3). Bars indicate standard errors. All experiments were done in three replicates, using two-way analysis of variance.

The efficacy of MbNPV against *E. onukii* increased over time following application, with no significant differences observed among the three concentrations (*p* > 0.05). Control efficacy reached approximately 90% by day 9 and 100% by day 21 for all concentrations (Figure 5a, Table S2). The population reduction rate followed a similar trend. On day 3, the reduction rates for the 900-fold (*p* = 0.04, t = 2.88) and 1400-fold (*p* = 0.04, t = 0.84) dilutions were significantly higher than the control, whereas the 400-fold dilution showed no significant difference from the control (*p* = 0.26, t = 2.03) (Figure 5b, Table S2). From day 5 to day 21, the population reduction rates for all three concentrations were significantly higher than the control (*p* < 0.001) (Figure 5b, Table S2).

### 3.4. Field Trial of CQMa421 at Different Concentrations Against E. onukii

**Figure 6:**
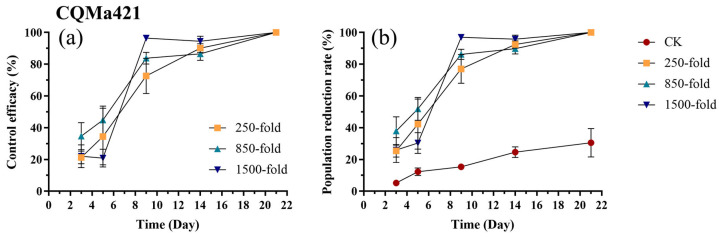
Control efficacy (**a**) and population reduction rate (**b**) of *E. onukii* following application of CQMa421 at different concentrations (250-, 850-, and 1500-fold). Data represent mean ± SE (n = 3). Bars indicate standard errors. All experiments were done in three replicates, using two-way analysis of variance.

Application of CQMa421, the control efficacy against *E. onukii* gradually increased, with no significant differences observed among different concentrations (*p* > 0.05). By day 9, all three concentrations exceeded 86%, and by day 21, all three reached 100% (Figure 6a, Table S3). The trend in the population reduction rate was similar to that of the control efficacy. On day 3, the population reduction rates for the 250-fold dilution (*p* = 0.12, t = 2.39) and the 1500-fold dilution (*p* = 0.11, t = 2.45) showed no significant differences compared to the control, while the 850-fold dilution resulted in a significantly higher population reduction rate than the control (*p* = 0.002, t = 3.867) (Figure 6b, Table S3). On day 5, the population reduction rates for the 250-fold dilution (*p* = 0.006, t = 3.54) and the 850-fold dilution (*p* < 0.001, t = 4.68) were significantly higher than the control, whereas the 1500-fold dilution showed no significant differences compared to the control (*p* = 0.21, t = 2.14) (Figure 6b, Table S3). From day 9 to day 21, the population reduction rates for all three concentrations were significantly higher than the control (*p* < 0.001) (Figure 6b, Table S3).

### 3.5. Field Trial of B. bassiana at Different Concentrations Against E. onukii

**Figure 7:**
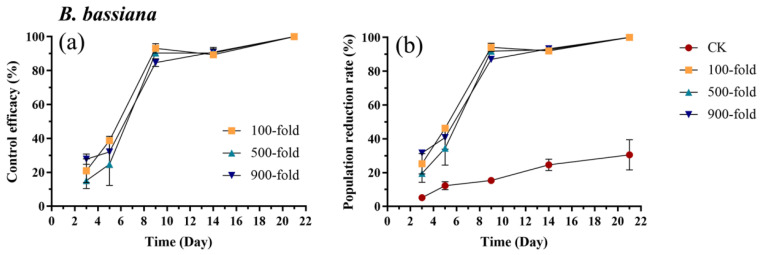
Control efficacy (**a**) and population reduction rate (**b**) of *E. onukii* following application of *B. bassiana* at different concentrations (100-, 500-, and 900-fold). Data represent mean ± SE (n = 3). Bars indicate standard errors. All experiments were done in three replicates, using two-way analysis of variance.

Application of *B. bassiana*, the control efficacy against *E. onukii* gradually increased, with no significant differences observed among different concentrations (*p* > 0.05). By day 9, all three concentrations exceeded 84%, and by day 21, all three reached 100% (Figure 7a, Table S4). The trend in the population reduction rate was similar to that of the control efficacy. On day 3, the population reduction rates for the 100-fold dilution (*p* = 0.008, t = 3.45) and the 900-fold dilution (*p* < 0.001, t = 4.55) were significantly higher than the control, while the 500-fold dilution showed no significant differences compared to the control (*p* = 0.10, t = 2.46). From day 5 to day 21, the population reduction rates for all three concentrations were significantly higher than the control (*p* < 0.001) (Figure 7b, Table S4).

### 3.6. Field Trial of Azadirachtin at Different Concentrations Against E. onukii

**Figure 8:**
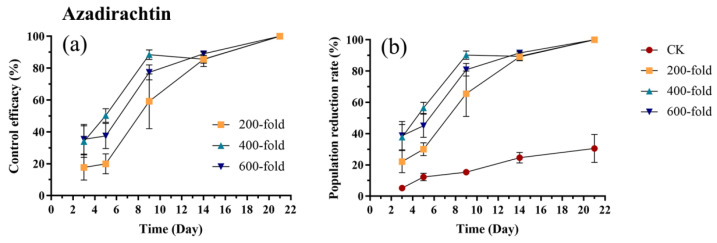
Control efficacy (**a**) and population reduction rate (**b**) of *E. onukii* following application of azadirachtin at different concentrations (200-, 400-, and 600-fold). Data represent mean ± SE (n = 3). Bars indicate standard errors. All experiments were done in three replicates, using two-way analysis of variance.

After the application of azadirachtin, the control efficacy against the *E. onukii* gradually increased. No significant differences were observed among different concentrations on days 3, 14, and 21 (*p* > 0.05). On days 5 and 9, the efficacy of the 200-fold dilution was significantly lower than that of the 400-fold dilution (Day 5: *p* = 0.01, t = 3.095; Day 9: *p* = 0.02, t = 2.976) (Figure 8a, Table S5). The trend in the population reduction rate was similar to that of the control efficacy. On day 3, the population reduction rates for the 400-fold dilution (*p* = 0.001, t = 4.08) and the 600-fold dilution (*p* < 0.001, t = 4.209) were significantly higher than the control, while the 200-fold dilution showed no significant differences compared to the control (*p* = 0.22, t = 2.12). On day 3, the population reduction rates for the 400-fold dilution (*p* < 0.001, t = 5.55) and the 600-fold dilution (*p* = 0.001, t = 4.11) were significantly higher than the control, while the 200-fold dilution showed no significant differences compared to the control (*p* = 0.18, t = 2.23). From day 9 to day 21, the population reduction rates for all three concentrations were significantly higher than the control (Figure 8b, Table S5).

### 3.7. Field Trial of Matrine at Different Concentrations Against E. onukii

**Figure 9:**
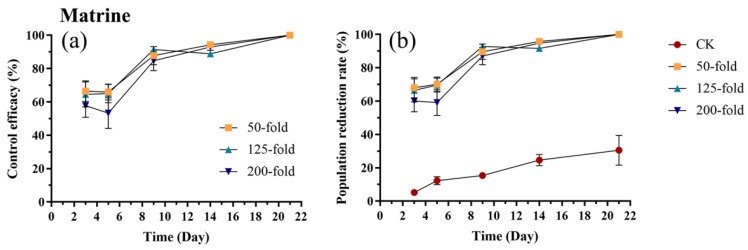
Control efficacy (**a**) and population reduction rate (**b**) of *E. onukii* following application of matrine at different concentrations (50-, 125-, and 200-fold). Data represent mean ± SE (n = 3). Bars indicate standard errors. All experiments were done in three replicates, using two-way analysis of variance.

Within three days of matrine application, control efficacy exceeded 50%, with the 50-fold dilution reaching 66.44% (Figure 9a). Efficacy for all three concentrations increased to 100% by day 21. From day 3 to day 21, no significant differences in efficacy were observed among different concentrations (*p* > 0.05) (Figure 9a, Table S6). The trend in the population reduction rate was similar to that of the control efficacy. The population reduction rates for all three concentrations were significantly higher than the control from day 3 to day 21 (*p* < 0.001) (Figure 9b, Table S6).

## 4. Discussion

This study identified seven feeding waveforms for *E. onukii*: Np, Pd, S, E, C, R, and F. These findings are consistent with previous reports on the closely related species *Empoasca vitis* by Miao et al. (2007) [42] and Zheng et al. (2017) [43]. Different nomenclature systems have been proposed for the feeding waveforms of the two tea leafhoppers, *E. onukii* and *E. vitis*. For example, Yorozuya (2016) [8] categorized the feeding waveforms of *E. onukii* into Np (non-probing phase, stylet not yet inserted into plant tissue); Eo1 (presumably a pathway exploration and channel construction phase, stylet inserted but no sap ingestion); Eo2 (presumably a phloem feeding phase, likely ingesting sap from the phloem); and Eo3 (presumably a non-phloem feeding phase, likely ingesting sap from mesophyll tissue). Jin et al. (2012) [7] classified the feeding waveforms of *E. vitis* into Np, E1, E2, E3, E4, E5, and E6. Among these, four waveforms (Np, E1, E2, and E3) were associated with probing and non-probing activities. E1 and all three of its waveform subtypes (E1-A to E1-C) were linked to the formation of a salivary sheath, stylet tearing, and channel cutting in viscous artificial diets. Both waveforms E2 and E3 were associated with active ingestion from liquid artificial diets. Most waveforms observed in this study can be mapped to those described in the aforementioned studies (Table 2). For instance, waveform Eo1 of *E. onukii* corresponds to waveform Pd in this study, Eo2 corresponds to waveform E, and Eo3 corresponds to waveform C.

The initiation of stylet probing is observed as waveform Np. This flat waveform indicates that the insect is not probing or that the stylet has not yet penetrated the leaf tissue. An increase in either the duration or frequency of waveform Np is positively correlated with the level of disruption to normal feeding behavior induced by the treatment [41]. In this study, treatments with MbNPV, CQMa421, and matrine significantly increased both the frequency and duration of waveform Np produced by adult *E. onukii* during probing (Figure 2b and Figure 3b). In contrast, azadirachtin significantly reduced the frequency of waveform Np but prolonged their duration (Figure 2b), indicating that all four aforementioned biopesticides possess feeding-interfering activity. All six biopesticides significantly reduced the frequency of waveforms S and Pd (Figure 2a,d,h) and shortened their duration (Figure 3a,d,h). Intracellular probing (waveform Pd) is the process by which the tea leafhopper penetrates leaf tissue, establishes channels, and damages multiple cells. It is primarily used to assess plant chemical composition and its palatability to the insect [41,43]. Waveform S is associated with saliva secretion and salivary sheath formation. *E. vitis* secretes gel-like saliva only during the initial probing phase, forming an incomplete salivary sheath that envelops the base of the stylet [7]. However, it is unclear whether *E. onukii* also forms a salivary sheath. In this study, waveform S often followed waveform pd, suggesting that the six biopesticides may interfere with *E. onukii* feeding by reducing the duration and frequency of channel cutting and salivary sheath formation.

Waveforms C and E represent the two primary feeding phases of E. onukii, corresponding to active ingestion when the stylet has not fully penetrated the phloem and passive ingestion when the stylet enters the phloem, respectively. Treatments with azadirachtin and B. bassiana significantly reduced the frequency of waveforms C and E (Figure 2c,e), indicating that these two biopesticides hinder sap-ingestion behavior of E. onukii. Previous studies have shown that the duration of waveform C is substantially shorter than that of waveform E in both duration and proportion, suggesting that sap-sucking insects primarily acquire nutrients via pressure-driven flow from the phloem into the stylets [8,43]. The results of this study appear to contradict this conclusion, as the duration and frequency of waveform C were roughly equivalent to, or even slightly higher than, those of waveform E. However, we observed that in the control group, E. onukii feeding was accompanied by mechanical obstruction (waveform F), which accounted for 9.45% of the total waveform events and 8.21% of the total duration (Figure 2a and Figure 3a). Waveform F frequently occurred before, between, or after waveforms C and E, suggesting that upon encountering a mechanical barrier, E. onukii is likely forced to abort ingestion and search for a new probing site. This conclusion is supported by Zheng et al. (2017) [43], who reported that the resistant tea cultivar ‘Huangguanyin’ exhibited the longest average duration of waveform F, whereas the susceptible cultivar ‘Tieguanyin’ showed the longest average duration of waveform E.

At the onset of waveform R, the stylets of *E. onukii* remain located in the phloem, which may represent either a buffering rest period during feeding obstruction or a slow withdrawal process of the stylets from the leaf tissue following prolonged hindered feeding. Consequently, waveforms R and F consistently increase or decrease simultaneously [43]. In this study, Bt, MbNPV, *B. bassiana*, and azadirachtin prolonged the duration of both R and F waveforms (Figure 3f,g), whereas MbNPV increased their frequency (Figure 2f,g). These findings align with the results reported by Hamouche et al. (2024) [41], who observed significantly longer total durations of waveform F in plants treated with Bt and *B. bassiana*. The disruption observed during the feeding process may be associated with pathogen-induced systemic resistance. The control function of Bt exhibits diversity, as both its spores and crystal proteins can induce broad-spectrum systemic immunity in tomatoes against multiple fungal diseases and pests [44]. *B. bassiana* induces resistance by colonizing plant tissues. For example, seed treatment with *B. bassiana* in the maize systemically enhances the activity of defense enzymes such as peroxidase (POD) and polyphenol oxidase (PPO) in leaves, thereby inhibiting the growth and development of pests like *Ostrinia furnacalis* [45].

Studies have found that aphid nitrogen acquisition primarily depends on a specific sustained feeding phase within the phloem (waveform E), whose successful establishment and maintenance are significantly influenced by host nitrogen levels. Specifically, aphids exhibit notably longer feeding durations in the high-nitrogen plants [46,47,48,49]. Furthermore, arbuscular mycorrhizal fungi (AMF) colonization is associated with reduced nitrogen content in leaves [46], suggesting that microbes may indirectly affect insects by modulating host nitrogen nutrition. Hence, we speculate that Bt and *B. bassiana* may similarly affect host plant nitrogen metabolism, potentially disrupting the establishment and maintenance of the insects’ passive phloem-feeding phase (waveform E). This disruption could, in turn, contribute to population decline, though further research is needed to confirm this hypothesis. In addition, piercing-sucking pests such as aphids primarily assess host plant nutritional quality by sensing amino acid concentrations in phloem sap, which determines whether they continue feeding [48]. Fungi can enhance proline content and the activity of multiple defense enzymes in plants, thereby improving plant resistance against pests [11]. Thus, fungal priming of plants may interfere with or influence early insect feeding behavior by altering the nutritional signals that insects rely on for host recognition. This sheds light on how entomopathogens may affect early host recognition and feeding in insect pests.

In this study, the control efficacy of fungi (*B. bassiana*, CQMa421), bacteria (Bt), and virus (MbNPV) increased rapidly to 84–90% during days 5–9 after application. This observation aligns with previous findings that within 5–7 days after fungal infection, the rapid proliferation of the fungus within host tissues and hemocoel (producing toxic compounds such as beauvericin that inhibit metabolic enzyme activity in insects) leads to complete paralysis of the infected insects [11]. Notably, matrine achieved 66.44% control efficacy as early as day 3, indicating its relatively rapid initial action. Studies of insect detoxification mechanisms further indicate a rise in glutathione-S-transferase activity during the initial three-day period after matrine application. This enzyme plays a major role in the detoxification of insecticides or pathogens [50,51]. Antioxidant enzyme activity rose initially but declined afterwards, weakening the insect’s capacity to scavenge reactive oxygen species, resulting in denaturation of various biomolecules and ultimately death [52,53].

In the present field trial, where the initial pest density was low to moderate (approximately 1–7 individuals/m^2^, Table S7), all six biopesticides demonstrated satisfactory control efficacy. During the early stage (3–9 days) following application, slight variations in efficacy were observed across different concentrations; however, these differences gradually narrowed over the treatment period. By day 21 post-application, all treatments (covering a wide range of concentrations, e.g., 100- to 350-fold for Bt; 50- to 200-fold for matrine) achieved 100% efficacy, with no significant differences among concentrations for most agents at the final time point. This suggests that lower concentrations within the tested ranges may be sufficient to achieve adequate control, particularly under low-to-moderate pest pressure. Therefore, this study supports the potential for dose optimization in practice, which could reduce application costs and environmental load while maintaining efficacy. Growers may consider using lower recommended concentrations, especially during early pest outbreaks, to enhance cost-effectiveness and sustainability.

It is important to acknowledge that field efficacy is influenced by multiple environmental variables, including weather conditions (temperature, humidity, rainfall) and the activity of natural enemies [54,55]. In field trials, applications were conducted under suitable weather conditions, and the observed pest population decline likely resulted from a combination of biopesticide action and natural mortality factors. Natural enemies, such as predatory spiders and parasitoid wasps, are known to play significant roles in regulating *E. onukii* populations in tea ecosystems [54,56], and it cannot be discounted that they contributed to the observed population decline, especially during the later stages of the trial [10]. Future studies should aim to quantify the relative contributions of biopesticides versus biotic and abiotic factors to better understand the mechanisms underlying field efficacy and to optimize application strategies under varying environmental conditions. Additionally, the present study did not directly measure tea yield parameters, which represents a limitation. Although the observed complete pest control and significant reduction in feeding activity suggest that treated plots likely experienced reduced damage and potentially higher yields compared to untreated controls, direct yield measurements would provide more concrete evidence of the agronomic and economic benefits of biopesticide application [57]. Therefore, future studies should incorporate comprehensive yield assessments alongside efficacy and behavioral evaluations to fully elucidate the practical value of biopesticides in tea production systems.

The six biopesticides represent four mechanistic classes with divergent target sites and behavioral disruption patterns: botanicals (azadirachtin and matrine) act primarily as rapid antifeedants [31,32,33,34,35], interfering with initial host recognition and active feeding; Bt functions as a stomach toxin that disrupts midgut physiology [36,37], thereby reducing passive phloem feeding; entomopathogenic fungi (*B. bassiana* and CQMa421) infect via cuticle penetration and proliferate within the hemocoel [38,39], causing systemic infection and prolonged resting behavior; and MbNPV induces viral pathogenesis with combined effects on both probing initiation and sustained feeding. Rotating these classes, rather than repeatedly using a single agent, reduces the likelihood of resistance evolution [10]. A practical rotation schedule could involve matrine or azadirachtin for rapid early-season knockdown when pests first appear, followed by Bt or *B. bassiana* during mid-season to disrupt feeding and maintain suppression; and MbNPV later in the season or during subsequent generations to introduce a composite mode of action. This approach not only preserves the efficacy of individual biopesticides but also aligns with IPM principles by integrating multiple control tactics. Future research should focus on validating optimal rotation sequences under field conditions and assessing their long-term impact on resistance dynamics, natural enemy communities, and overall tea ecosystem health.

While this study focused on evaluating the efficacy of six biopesticides against *E. onukii*, it is instructive to consider how these biopesticides compare with conventional chemical pesticides commonly used in tea plantations, such as bifenthrin, chlorfenapyr, and thiamethoxam [9,10]. Chemical pesticides typically exhibit rapid knock-down effects, often achieving > 90% control within 24–72 h post-application. However, they are associated with significant drawbacks, including the development of pesticide resistance, resurgence of secondary pests, negative impacts on natural enemy populations, and concerns over chemical residues in harvested tea [9,10]. In contrast, the biopesticides evaluated in this study demonstrated a slower but progressive mode of action, with control efficacy reaching 84–90% by days 5–9 and 100% by day 21 post-application. Although their initial speed of kill is slower than synthetic chemicals, biopesticides offer distinct advantages in terms of environmental safety, compatibility with biological control agents, and suitability for organic and residue-free tea production [10]. Furthermore, their ability to disrupt feeding behavior within hours (as revealed by EPG analysis) means that feeding damage is reduced almost immediately, even before mortality occurs. Regarding residual activity, biopesticides generally have shorter environmental persistence compared to synthetic chemicals, which can be both an advantage (reduced environmental contamination) and a limitation (potentially requiring more precise timing or repeated applications) [10]. Future research should directly compare biopesticides and conventional products under identical field conditions to quantify trade-offs in speed of action, persistence, cost-effectiveness, and overall compatibility with sustainable tea production goals.

## 5. Conclusions

In summary, all six tested biopesticides were able to disrupt the feeding behavior of *E. onukii* within a short period. Two botanical pesticides (azadirachtin and matrine) interfered primarily by prolonging the duration or increasing the frequency of non-feeding periods (waveform Np) while reducing both the duration and frequency of the active non-phloem feeding phase (waveform C). Bt extended the duration of the resting phase (waveform R) and decreased the frequency of passive phloem feeding (waveform E). The two entomopathogenic fungi, CQMa421 and *B. bassiana*, acted through slightly different mechanisms: CQMa421 prolonged waveform Np but reduced waveform C in both duration and frequency, resembling the botanical pesticides; *B. bassiana* increased the duration and frequency of waveform R and reduced waveform E frequency, similar to Bt. MbNPV not only extended the duration and frequency of both waveform Np and waveform R but also decreased the duration and frequency of waveform C. Beyond causing early feeding disruption, all six biopesticides also demonstrated effective field control of *E. onukii* under low-density infestation conditions. This study reveals the specific mechanisms by which different types of biopesticides interfere with pest feeding behavior, provides promising candidates for the green control of *E. onukii* in tea plantations, and highlights their significance for promoting safe tea production and ecologically friendly pest management.

## Figures and Tables

**Table 1:** Dilution ratios of six biopesticides.

Full Name (Abbreviation)	*Bacillus thuringiensis* (Bt)	0.3% Azadirachtin EC (Azadirachtin)	*Metarhizium anisopliae* Strain CQMa421 OD with 8 × 10^8^ Spores/mL (CQMa421)	2.0% Matrine OD (Matrine)	*Mamestra brassicae* Nucleopolyhedrovirus (MbNPV)	*Beauveria bassiana* WP with 3 × 10^10^ Spores/g (*Beauveria bassiana*)
Dilution factor	100	200	250	50	400	150
	250	400	850	125	900	500
	350	600	1500	175	1400	900

**Table 2:** Comparative nomenclature of *Empoasca* feeding waveforms across studies.

Waveform of *E. onukii* on Tea Plants in This Study	Waveform of *E. onukii* on Tea Plants [8]	Waveform of *E. vitis* on Liquid Diet [7]	Biological Significance
Np	Np	Np	Non-probing
Pd	Eo1	E1	Probing
C	Eo3	E2\E3	Active feeding (non-phloem feeding phase)
E	Eo2	-	Passive feeding (phloem feeding phase)
R	-	-	Resting
F	-	-	Mechanical obstruction
S	-	E1	Salivary secretion

## Data Availability

Data will be made available upon request.

## Supplementary Materials

The following supporting information can be downloaded at: https://www.mdpi.com/article/10.3390/biology15050419/s1, Table S1. Statistical data of Bt field trials; Table S2. Statistical data of MbNPV field trials; Table S3. Statistical data of CQMa421 field trials; Table S4. Statistical data of *Beauveria bassiana* field trials; Table S5. Statistical data of azadirachtin field trials; Table S6. Statistical data of matrine field trials; Table S7. Raw data of the field trial.

## Abbreviations

The following abbreviations are used in this manuscript:

EPG	Electrical penetration graph
MbNPV	Mamestra brassicae nucleopolyhedrovirus

## References

[1] Deka B., Babu A., Pandey A.K., Kumhar K.C., Rajbongshi H., Dey P., Peter A.J., Amalraj E.L.D., Talluri V.R. (2022). Potential of the entomopathogenic fungus, *Metarhizium anisopliae* s. l. for control of red spider mite, *Oligonychus coffeae* Nietner on tea crop. Int. J. Acarol..

[2] Pokharel S.S., Yu H., Fang W., Parajulee M.N., Chen F. (2023). Intercropping cover crops for a vital ecosystem service: A review of the biocontrol of insect pests in tea agroecosystems. Plants.

[3] Hazarika L.K., Bhuyan M., Hazarika B.N. (2009). Insect pests of tea and their management. Annu. Rev. Entomol..

[4] Kumar J., Shakil N.A., Khan M.A., Malik K., Walia S. (2011). Development of controlled release formulations of carbofuran and imidacloprid and their bioefficacy evaluation against aphid, *Aphis gossypii* and leafhopper, *Amrasca biguttula biguttula* Ishida on potato crop. J. Environ. Sci. Health Part B.

[5] Chen K., Huang M.X., Shi Q.C., Xie X., Jin L.H., Xu W.M., Li X.Y. (2019). Screening of a potential leafhopper attractants and their applications in tea plantations. J. Environ. Sci. Health Part B.

[6] Backus E.A., Serrano M.S., Ranger C.M. (2005). Mechanisms of hopperburn: An overview of insect taxonomy, behavior and physiology. Annu. Rev. Entomol..

[7] Jin S., Chen Z.M., Backus E.A., Sun X.L., Xiao B. (2012). Characterization of EPG waveforms for the tea green leafhopper, *Empoasca vitis* Göthe (Hemiptera: Cicadellidae), on tea plants and their correlation with stylet activities. J. Insect Physiol..

[8] Yorozuya H. (2016). Monitoring and characterization of DC electrical penetration graph waveforms of tea green leafhopper, *Empoasca onukii*, on tea plants. Entomol. Sci..

[9] Zhao Y., Song Q., Song Y. (2023). The role of insect intestinal microbes in controlling of *Empoasca onukii* Matsuda (Hemiptera: Cicadellidae) pest infestations in the production of tea garden: A review. Arch. Microbiol..

[10] Chen Z., Luo Z. (2025). Management of insect pests on tea plantations: Safety, sustainability, and efficiency. Annu. Rev. Entomol..

[11] Bhattacharyya P.N., Sarmah S.R., Roy S., Sarma B., Nath B.C., Bhattacharyya L.H. (2023). Perspectives of *Beauveria bassiana*, an entomopathogenic fungus for the control of insect-pests in tea [*Camellia sinensis* (L.) O. Kuntze]: Opportunities and challenges. Int. J. Trop. Insect Sci..

[12] Kumhar K.C., Babu A., Arulmarianathan J.P., Deka B., Bordoloi M., Rajbongshi H., Dey P. (2020). Role of beneficial fungi in managing diseases and insect pests of tea plantation. Egypt. J. Biol. Pest Control.

[13] Roy S., Prasad A.K., Neave S., Bhattacharyya P.N., Nagpal A., Borah K., Rahman A., Sarmah M., Sarmah S.R., Pandit V. (2020). Nonchemical based integrated management package for live-wood eating termites in tea plantations of north-east India. Int. J. Trop. Insect Sci..

[14] Deka B., Babu A., Peter A.J., Pandey A.K., Kumhar K.C., Sarkar S., Rajbongshi H., Dey P., Amalraj E.L., Talluri V.R. (2021). *Beauveria bassiana*: As a potential microbial biocontrol agent for tea mosquito bug, *Helopeltis theivora* Waterhouse (Hemiptera: Miridae) in Dooars and Darjeeling, India. Egypt. J. Biol. Pest Control.

[15] Deka B., Babu A. (2021). Tea pest management: A microbiological approach. Appl. Microbiol..

[16] Qadri M., Short S., Gast K., Hernandez J., Wong A.C.N. (2020). Microbiome innovation in agriculture: Development of microbial based tools for insect pest management. Front. Sustain. Food Syst..

[17] Singha D., Singha B., Dutta B.K. (2011). Potential of *Metarhizium anisopliae* and *Beauveria bassiana* in the control of tea termite *Microtermes obesi* Holmgren in vitro and under field conditions. J. Pest Sci..

[18] Dashora K., Roy S., Nagpal A., Roy S.M., Flood J., Prasad A.K., Khetarpal R., Neave S., Muraleedharan N. (2017). Pest management through *Bacillus thuringiensis* (Bt) in a tea-silkworm ecosystem: Status and potential prospects. Appl. Microbiol. Biotechnol..

[19] Antony B., Sinu P.A., Das S. (2011). New record of nucleopolyhedroviruses in tea looper caterpillars in India. J. Invertebr. Pathol..

[20] Zhang X., Wang Y., Yi J., Guan S.X., Jiang J.X., Qiao Y.Y., Chen Q.X., Liu S., Huang J.G., Liu Y.F. (2021). Evaluation of the efficacy of four insecticides against geometridae in tea gardens. Biol. Disaster Sci..

[21] Zhan J., Zhang A., Deng F., Lu S., Sun X. (2020). Application and promotion of *Mamestra brassicae* nucleopolyhedrovirus suspension forcontrol of *Spodoptera frugiperda*. Chin. J. Biol. Control.

[22] Zhang X.X., Mei Y., Li H., Tang M.J., He K., Tang M.J., He K., Xiao Q. (2021). Differences in virulence and genomics of two *Ectropis obliqua* nucleopolyhedrovirus strains to *Ectropis grisescens*. J. Plant Prot..

[23] Liu Y., Zhou Z., Zhang M., Wei W., He S., Wang M., Yu C., Ma F., Che A. (2025). Laboratory insecticidal effect determination and field control efficacy evaluation of different insecticides against *Tuta absoluta*. J. Cold-Arid Agric. Sci..

[24] Anitha S., Mahendran P., Selvakumar S., Janarthanan P., Raghunath M., Megala R., Vinitha Ebziba C., Vidya S.L., Sagadevan P. (2019). Bio-efficacy of entomopathogenic fungus *Metarhizium anisopliae* (METSCH) against the tea mosquito bug, *Helopeltis theivora* (Waterhouse) and the red spider mite, *Oligonychus coffeae* (Nietner) infecting tea in south India. Int. J. Entomol. Res..

[25] Cheramgoi E., Wanjala F.M.E., Sudoi V., Wanyoko J., Mwamburi L., Nyukuri R. (2016). Efficacy and mode of application of local *Beauveria bassiana* isolates in the control of the tea weevil. Annu. Res. Rev. Biol..

[26] Peng G., Zhang S., Xia Y. (2020). *Metarhizium anisopliae* CQMa421 and its application status. Chin. J. Biol. Control.

[27] De França S.M., Breda M.O., Barbosa D.R., Araujo A.M., Guedes C.A., Shields V.D.C. (2017). The sublethal effects of insecticides in insects. Biol. Control Pest Vector Insects.

[28] Müller C. (2018). Impacts of sublethal insecticide exposure on insects—Facts and knowledge gaps. Basic Appl. Ecol..

[29] Koul O. (2008). Phytochemicals and insect control: An antifeedant approach. Crit. Rev. Plant Sci..

[30] Garzo E., Moreno A., Plaza M., Fereres A. (2020). Feeding behavior and virus-transmission ability of insect vectors exposed to systemic insecticides. Plants.

[31] Roy S., Mukhopadhyay A., Gurusubramanian G. (2010). Field efficacy of a biopesticide prepared from *Clerodendrum viscosum* Vent. (Verbenaceae) against two major tea pests in the sub Himalayan tea plantation of North Bengal, India. J. Pest Sci..

[32] Vasanthakumar D., Babu A., Shanmugapriyan R., Subramaniam S.R. (2013). Impact of Azter (azadirachtin 0.15% EC), a neem-based pesticide, against tea red spider mite, *Oligonychus coffeae* Nietner (Acarina: Tetranychidae), and its natural enemies. Int. J. Acarol..

[33] Sola P., Mvumi B.M., Ogendo J.O., Mponda O., Kamanula J.F., Nyirenda S.P., Belmain S.R., Stevenson P.C. (2014). Botanical pesticide production, trade and regulatory mechanisms in sub-Saharan Africa: Making a case for plant-based pesticidal products. Food Secur..

[34] Roy S., Muraleedharan N., Mukhapadhyay A., Handique G. (2015). The tea mosquito bug, *Helopeltis theivora* Waterhouse (Heteroptera: Miridae): Its status, biology, ecology and management in tea plantations. Int. J. Pest Manag..

[35] Tian Y.Y., Chen Z.J., Huang X.Q., Zhang L.X., Zhang Z.Q. (2020). Evaluation of botanicals for management of piercing–sucking pests and the effect on beneficial arthropod populations in tea trees *Camellia sinensis* (L.) O. Kuntze (Theaceae). J. Insect Sci..

[36] Gutiérrez M.E.M., Capalbo D.M.F., de Oliveira Arruda R., de Oliveira Moraes R., Souza B., Vázquez L., Marucci R. (2019). *Bacillus* *thuringiensis*. Natural Enemies of Insect Pests in Neotropical Agroecosystems.

[37] Bah A., van Frankenhuyzen K., Brousseau R., Masson L. (2004). The *Bacillus thuringiensis* Cry1Aa toxin: Effects of trypsin and chymotrypsin site mutations on toxicity and stability. J. Invertebr. Pathol..

[38] Wang H., Peng H., Li W., Cheng P., Gong M. (2021). The toxins of *Beauveria bassiana* and the strategies to improve their virulence to insects. Front. Microbiol..

[39] Song S., Li S., Luo Y., Wei D., Shang J., Wu H., Fang G., Wang C. (2025). Dual disruption of the immune cytokine Spätzle facilitates fungal infection of diverse insect hosts. Adv. Sci..

[40] Hong S., Sun Y., Chen H., Wang C. (2023). Suppression of the insect cuticular microbiomes by a fungal defensin to facilitate parasite infection. ISME J..

[41] Hamouche Z., Zippari C., Boucherf A., Cavallo G., Djelouah K., Tamburini G., Verrastro V., Biondi A., Cornara D. (2024). Impact of biopesticides on the probing and feeding behavior of *Aphis gossypii*. CABI Agric. Biosci..

[42] Miao J., Han B. (2007). The probing behavior of the tea green leafhopper on different tea plant cultivars. Acta Ecol. Sin..

[43] Zheng Y., Wang M., Cui L., Han S., Yu P., Han B. (2017). Resistance of tea cultivars to the tea green leafhopper analyzed by EPG technique and their resistance-related substances. Acta Ecol. Sin..

[44] Gupta R., Keppanan R., Leibman-Markus M., Matveev S., Rav-David D., Shulhani R., Elad Y., Ment D., Bar M. (2024). *Bacillus thuringiensis* promotes systemic immunity in tomato, controlling pests and pathogens and promoting yield. Food Secur..

[45] Zhan Z., Gou X., Wang Z., Zhang Y., Wang X., Guo J. (2025). Effects of seed soaking with *Beauveria bassiana* and *Metarhizium rileyi* on defense enzyme activities and insect resistance in maize leaves. Chin. J. Biol. Control.

[46] Ponder K.L., Pritchard J., Harrington R., Bale J.S. (2000). Difficulties in location and acceptance of phloem sap combined with reduced concentration of phloem amino acids explain lowered performance of the aphid *Rhopalosiphum padi* on nitrogen deficient barley (*Hordeum vulgare*) seedlings. Entomol. Exp. Et Appl..

[47] Bezemer T.M., De Deyn G.B., Bossinga T.M., Van Dam N.M., Harvey J.A., Van Der Putten W.H. (2005). Soil community composition drives aboveground plant–herbivore–parasitoid interactions. Ecol. Lett..

[48] Nowak H., Komor E. (2010). How aphids decide what is good for them: Experiments to test aphid feeding behaviour on *Tanacetum vulgare* (L.) using different nitrogen regimes. Oecologia.

[49] Kuhlmann F., Opitz S.E.W., Inselsbacher E., Ganeteg U., Nasholm T., Ninkovic V. (2013). Exploring the nitrogen ingestion of aphids—A new method using electrical penetration graph and N-15 labelling. PLoS ONE.

[50] Claudianoc C., Ranson H., Johnson R.M., Biswas S., Schuler M.A., Berenbaum M.R., Feyereisen R., Oakeshott J.G. (2006). A deficit of detoxification enzymes: Pesticide sensitivity and environmental response in the honeybee. Insect Mol. Biol..

[51] Jia M., Cao M., Li Y., Tu X., Wang G., Nong X., Whitman D.W., Zhang Z. (2016). Biochemical basis of synergism between pathogenic fungus *Metarhizium anisopliae* and insecticide chlorantraniliprole in *Locusta migratoria* (Meyen). Sci. Rep..

[52] Felton G.W., Summers C.B. (1995). Antioxidant systems in insects. Arch. Insect Biochem. Physiol..

[53] Wu J., Li J., Zhang C., Yu X., Cuthbertson A.G., Ali S. (2020). Biological impact and enzyme activities of *Spodoptera litura* (Lepidoptera: Noctuidae) in response to synergistic action of matrine and *Beauveria brongniartii*. Front. Physiol..

[54] Chen L.L., Yuan P., Pozsgai G., Chen P., Zhu H., You M.S. (2019). The impact of cover crops on the predatory mite *Anystis baccarum* (Acari, Anystidae) and the leafhopper pest *Empoasca onukii* (Hemiptera, Cicadellidae) in a tea plantation. Pest Manag. Sci..

[55] Han H., Zou K., Yuan Z. (2024). Impact of specialized agricultural services on climate-smart agricultural practices: Evidence from biopesticide application in Jiangsu Province, China. Environ. Impact Assess. Rev..

[56] Yang T., Song X., Zhong Y., Wang B., Zhou C. (2022). Field investigation- and dietary metabarcoding-based screening of arthropods that prey on primary tea pests. Ecol. Evol..

[57] Piyasena K.G.N.P., Hettiarachchi L.S.K. (2023). Comparison of tea quality parameters of conventionally and organically grown tea, and effects of fertilizer on tea quality: A mini-review. Food Chem. Adv..

