# Supplementary material for: Short-Term Feeding Disruption Effects and Efficacy of Six Biopesticides Against Empoasca onukii (Hemiptera: Cicadellidae)
Source: Biology (Basel). 2026 Mar 4;15(5):419. doi: 10.3390/biology15050419 (PMC12984874; doi:10.3390/biology15050419)
Supplement: Supplementary file 1 [file biology-15-00419-s001.zip › biology-4159327-supplementary.pdf]

Supplementary information

Table S1. Statistical data of Bt field trials

Time	Population reduction rate						Control efficacy					
		Mean Diff.	95.00% CI of diff.	Summary	P Value	t		Mean Diff.	95.00% CI of diff.	Summary	Adjusted P Value	t
3d	100-fold vs. CK	16.98	1.904 to 32.06	*	0.02	3.117	100-fold vs. 250-fold	-9.135	-24.19 to 5.921	ns	0.35	1.534
	250-fold vs. CK	25.40	10.33 to 40.48	***	<0.001	4.663	100-fold vs. 350-fold	0.6389	-14.42 to 15.70	ns	>0.99	0.1073
	350-fold vs. CK	16.31	1.234 to 31.39	*	0.03	2.994	250-fold vs. 350-fold	9.774	-5.282 to 24.83	ns	0.30	1.641
5d	100-fold vs. CK	33.25	18.17 to 48.33	***	<0.001	6.104	100-fold vs. 250-fold	-21.52	-36.57 to -6.461	**	0.003	3.614
	250-fold vs. CK	52.48	37.41 to 67.56	***	<0.001	9.635	100-fold vs. 350-fold	3.101	-11.96 to 18.16	ns	0.94	0.5208
	350-fold vs. CK	31.06	15.99 to 46.14	***	<0.001	5.702	250-fold vs. 350-fold	24.62	9.562 to 39.68	***	<0.001	4.134
9d	100-fold vs. CK	78.53	63.46 to 93.61	***	<0.001	14.42	100-fold vs. 250-fold	-0.3319	-15.39 to 14.72	ns	>0.99	0.05573

	250-fold vs. CK	78.92	63.84 to 94.00	***	<0.001	14.49	100-fold vs. 350-fold	4.901	-10.16 to 19.96	ns	0.80	0.8230
	350-fold vs. CK	74.48	59.40 to 89.55	***	<0.001	13.67	250-fold vs. 350-fold	5.233	-9.824 to 20.29	ns	0.77	0.8787
	100-fold vs. CK	71.61	56.53 to 86.68	***	<0.001	13.15	100-fold vs. 250-fold	24.73	9.678 to 39.79	***	<0.001	4.154
14d	250-fold vs. CK	53.27	38.20 to 68.35	***	<0.001	9.780	100-fold vs. 350-fold	-0.1919	-15.25 to 14.86	ns	>0.99	0.03222
	350-fold vs. CK	71.69	56.61 to 86.76	***	<0.001	13.16	250-fold vs. 350-fold	-24.93	-39.98 to -9.870	***	<0.001	4.186
	100-fold vs. CK	69.46	54.39 to 84.54	***	<0.001	12.75	100-fold vs. 250-fold	0.000	-15.06 to 15.06	ns	>0.99	0.000
21d	250-fold vs. CK	69.46	54.39 to 84.54	***	<0.001	12.75	100-fold vs. 350-fold	0.000	-15.06 to 15.06	ns	>0.99	0.000
	350-fold vs. CK	69.46	54.39 to 84.54	***	<0.001	12.75	250-fold vs. 350-fold	0.000	-15.06 to 15.06	ns	>0.99	0.000

Table S2. Statistical data of MbNPV field trials

Time	Population reduction rate						Control efficacy					
		Mean Diff.	95.00% CI of diff.	Summary	Adjusted P Value	t		Mean Diff.	95.00% CI of diff.	Summary	Adjusted P Value	t
3d	CK vs. 400-fold	-14.44	-34.11 to 5.242	ns	0.26	2.030	400-fold vs. 900-fold	-6.319	-28.21 to 15.57	ns	0.85	0.7300
	CK vs. 900-fold	-20.46	-40.14 to -0.7816	*	0.04	2.878	400-fold vs. 1400-fold	-6.147	-28.04 to 15.74	ns	0.86	0.7101
	CK vs. 1400-fold	-20.19	-39.87 to -0.5127	*	0.04	2.840	900-fold vs. 1400-fold	0.1716	-21.72 to 22.06	ns	>0.99	0.01983
5d	CK vs. 400-fold	-44.32	-64.00 to -24.65	***	<0.001	6.234	400-fold vs. 900-fold	0.1351	-21.75 to 22.02	ns	>0.99	0.01561
	CK vs. 900-fold	-44.91	-64.58 to -25.23	***	<0.001	6.316	400-fold vs. 1400-fold	18.88	-3.005 to 40.77	ns	0.11	2.181
	CK vs. 1400-fold	-27.49	-47.17 to -7.810	**	0.002	3.866	900-fold vs. 1400-fold	18.75	-3.140 to 40.64	ns	0.11	2.166
9d	CK vs. 400-fold	-74.67	-94.35 to -55.00	***	<0.001	10.50	400-fold vs. 900-fold	-3.013	-24.90 to 18.87	ns	0.98	0.3481
	CK vs. 900-fold	-77.29	-96.97 to -57.61	***	<0.001	10.87	400-fold vs. 1400-fold	1.041	-20.85 to 22.93	ns	>0.99	0.1202

	CK vs. 1400-fold	-73.80	-93.48 to -54.12	***	<0.001	10.38	900-fold vs. 1400-fold	4.054	-17.83 to 25.94	ns	0.95	0.4683
14d	CK vs. 400-fold	-64.40	-84.08 to -44.72	***	<0.001	9.058	400-fold vs. 900-fold	-1.620	-23.51 to 20.27	ns	>0.99	0.1872
	CK vs. 900-fold	-65.58	-85.26 to -45.90	***	<0.001	9.223	400-fold vs. 1400-fold	2.831	-19.06 to 24.72	ns	0.98	0.3271
	CK vs. 1400-fold	-62.46	-82.14 to -42.78	***	<0.001	8.785	900-fold vs. 1400-fold	4.451	-17.44 to 26.34	ns	0.94	0.5142
21d	CK vs. 400-fold	-69.46	-89.14 to -49.79	***	<0.001	9.770	400-fold vs. 900-fold	0.000	-21.89 to 21.89	ns	>0.99	0.000
	CK vs. 900-fold	-69.46	-89.14 to -49.79	***	<0.001	9.770	400-fold vs. 1400-fold	0.000	-21.89 to 21.89	ns	>0.99	0.000
	CK vs. 1400-fold	-69.46	-89.14 to -49.79	***	<0.001	9.770	900-fold vs. 1400-fold	0.000	-21.89 to 21.89	ns	>0.99	0.000

Table S3. Statistical data of CQMa421 field trials

Time	Population reduction rate						Control efficacy					
		Mean Diff.	95.00% CI of diff.	Summary	Adjusted P Value	t		Mean Diff.	95.00% CI of diff.	Summary	Adjusted P Value	t
3d	CK vs. 250-fold	-20.19	-43.58 to 3.207	ns	0.12	2.388	250-fold vs. 850-fold	-13.44	-38.56 to 11.68	ns	0.46	1.353
	CK vs. 850-fold	-32.68	-56.08 to -9.293	**	0.002	3.867	250-fold vs. 1500-fold	-0.7845	-25.90 to 24.33	ns	>0.99	0.07897
	CK vs. 1500-fold	-20.72	-44.12 to 2.668	ns	0.11	2.452	850-fold vs. 1500-fold	12.65	-12.46 to 37.77	ns	0.51	1.274
5d	CK vs. 250-fold	-29.96	-53.35 to -6.566	**	0.006	3.544	250-fold vs. 850-fold	-10.42	-35.54 to 14.70	ns	0.66	1.049
	CK vs. 850-fold	-39.51	-62.91 to -16.12	***	<0.001	4.675	250-fold vs. 1500-fold	13.60	-11.52 to 38.72	ns	0.45	1.369
	CK vs. 1500-fold	-18.07	-41.46 to 5.321	ns	0.21	2.138	850-fold vs. 1500-fold	24.02	-1.098 to 49.14	ns	0.06	2.418
9d	CK vs. 250-fold	-61.68	-85.07 to -38.29	***	<0.001	7.298	250-fold vs. 850-fold	-11.15	-36.26 to 13.97	ns	0.61	1.122
	CK vs. 850-fold	-70.85	-94.24 to -47.45	***	<0.001	8.382	250-fold vs. 1500-fold	-23.78	-48.90 to 1.337	ns	0.07	2.394

	CK vs. 1500-fold	-81.59	-105.0 to -58.20	***	<0.001	9.654	850-fold vs. 1500-fold	-12.63	-37.75 to 12.48	ns	0.51	1.272
	CK vs. 250-fold	-67.83	-91.22 to -44.44	***	<0.001	8.025	250-fold vs. 850-fold	3.634	-21.48 to 28.75	ns	0.98	0.3658
14d	CK vs. 850-fold	-65.18	-88.58 to -41.79	***	<0.001	7.712	250-fold vs. 1500-fold	-4.299	-29.42 to 20.82	ns	0.96	0.4327
	CK vs. 1500-fold	-71.16	-94.55 to -47.77	***	<0.001	8.419	850-fold vs. 1500-fold	-7.932	-33.05 to 17.19	ns	0.82	0.7985
	CK vs. 250-fold	-69.46	-92.86 to -46.07	***	<0.001	8.219	250-fold vs. 850-fold	0.000	-25.12 to 25.12	ns	>0.99	0.000
21d	CK vs. 850-fold	-69.46	-92.86 to -46.07	***	<0.001	8.219	250-fold vs. 1500-fold	0.000	-25.12 to 25.12	ns	>0.99	0.000
	CK vs. 1500-fold	-69.46	-92.86 to -46.07	***	<0.001	8.219	850-fold vs. 1500-fold	0.000	-25.12 to 25.12	ns	>0.99	0.000

Table S4. Statistical data of *Beauveria bassiana* field trials

Time	Population reduction rate						Control efficacy					
		Mean Diff.	95.00% CI of diff.	Summary	P Value	t		Mean Diff.	95.00% CI of diff.	Summary	Adjusted P Value	t
3d	CK vs. 100-fold	-20.00	-36.06 to -3.949	**	0.008	3.448	100-fold vs. 500-fold	5.817	-11.55 to 23.19	ns	0.79	0.8467
	CK vs. 500-fold	-14.28	-30.33 to 1.777	ns	0.10	2.461	100-fold vs. 900-fold	-6.810	-24.18 to 10.56	ns	0.70	0.9913
	CK vs. 900-fold	-26.42	-42.47 to -10.36	***	<0.001	4.554	500-fold vs. 900-fold	-12.63	-30.00 to 4.743	ns	0.21	1.838
5d	CK vs. 100-fold	-33.97	-50.03 to -17.92	***	<0.001	5.856	100-fold vs. 500-fold	13.85	-3.521 to 31.22	ns	0.15	2.016
	CK vs. 500-fold	-22.14	-38.20 to -6.088	**	0.003	3.817	100-fold vs. 900-fold	6.637	-10.73 to 24.01	ns	0.71	0.9661
	CK vs. 900-fold	-28.42	-44.48 to -12.37	***	<0.001	4.900	500-fold vs. 900-fold	-7.213	-24.58 to 10.16	ns	0.66	1.050
9d	CK vs. 100-fold	-78.72	-94.77 to -62.66	***	<0.001	13.57	100-fold vs. 500-fold	2.751	-14.62 to 20.12	ns	2.751	0.4005
	CK vs. 500-fold	-76.47	-92.52 to -60.41	***	<0.001	13.18	100-fold vs. 900-fold	8.398	-8.972 to 25.77	ns	8.398	1.223

	CK vs. 900-fold	-71.66	-87.71 to -55.60	***	<0.001	12.35	500-fold vs. 900-fold	5.647	-11.72 to 23.02	ns	5.647	0.8220
	CK vs. 100-fold	-67.42	-83.48 to -51.37	***	<0.001	11.62	100-fold vs. 500-fold	-0.9911	-18.36 to 16.38	ns	>0.99	0.1443
14d	CK vs. 500-fold	-68.14	-84.19 to -52.08	***	<0.001	11.75	100-fold vs. 900-fold	-1.512	-18.88 to 15.86	ns	>0.99	0.2201
	CK vs. 900-fold	-68.72	-84.77 to -52.66	***	<0.001	11.85	500-fold vs. 900-fold	-0.5208	-17.89 to 16.85	ns	>0.99	0.07581
	CK vs. 100-fold	-69.46	-85.52 to -53.41	***	<0.001	11.97	100-fold vs. 500-fold	0.000	-17.37 to 17.37	ns	>0.99	0.000
21d	CK vs. 500-fold	-69.46	-85.52 to -53.41	***	<0.001	11.97	100-fold vs. 900-fold	0.000	-17.37 to 17.37	ns	>0.99	0.000
	CK vs. 900-fold	-69.46	-85.52 to -53.41	***	<0.001	11.97	500-fold vs. 900-fold	0.000	-17.37 to 17.37	ns	>0.99	0.000

Table S5. Statistical data of azadirachtin field trials

Time	Population reduction rate						Control efficacy					
		Mean Diff.	95.00% CI of diff.	Summary	Adjusted P Value	t		Mean Diff.	95.00% CI of diff.	Summary	Adjusted P Value	t
3d	CK vs. 200-fold	-16.89	-38.92 to 5.129	ns	0.22	2.123	200-fold vs. 400-fold	-16.24	-41.07 to 8.594	ns	0.29	1.653
	CK vs. 400-fold	-32.49	-54.51 to -10.47	**	0.001	4.083	200-fold vs. 600-fold	-17.66	-42.49 to 7.166	ns	0.23	1.799
	CK vs. 600-fold	-33.49	-55.52 to -11.47	***	<0.001	4.209	400-fold vs. 600-fold	-1.428	-26.26 to 23.40	ns	>0.99	0.1455
5d	CK vs. 200-fold	-17.72	-39.74 to 4.307	ns	0.18	2.226	200-fold vs. 400-fold	-30.39	-55.22 to -5.564	*	0.01	3.095
	CK vs. 400-fold	-44.16	-66.18 to -22.14	***	<0.001	5.550	200-fold vs. 600-fold	-17.50	-42.33 to 7.335	ns	0.23	1.782
	CK vs. 600-fold	-32.69	-54.71 to -10.66	**	0.001	4.108	400-fold vs. 600-fold	12.90	-11.93 to 37.73	ns	0.49	1.314
9d	CK vs. 200-fold	-50.16	-72.18 to -28.13	***	<0.001	6.303	200-fold vs. 400-fold	-29.22	-54.05 to -4.390	*	0.02	2.976
	CK vs. 400-fold	-74.80	-96.82 to -52.78	***	<0.001	9.400	200-fold vs. 600-fold	-18.16	-42.99 to 6.671	ns	0.21	1.849

	CK vs. 600-fold	-65.46	-87.48 to -43.44	***	<0.001	8.227	400-fold vs. 600-fold	11.06	-13.77 to 35.89	ns	0.61	1.126
14d	CK vs. 200-fold	-64.49	-86.51 to -42.47	***	<0.001	8.104	200-fold vs. 400-fold	-0.01963	-24.85 to 24.81	ns	>0.99	0.001999
	CK vs. 400-fold	-64.83	-86.85 to -42.80	***	<0.001	8.147	200-fold vs. 600-fold	-3.462	-28.29 to 21.37	ns	0.98	0.3526
	CK vs. 600-fold	-67.05	-89.08 to -45.03	***	<0.001	8.427	400-fold vs. 600-fold	-3.443	-28.27 to 21.39	ns	0.98	0.3506
21d	CK vs. 200-fold	-69.46	-91.49 to -47.44	***	<0.001	8.730	200-fold vs. 400-fold	0.000	-24.83 to 24.83	ns	>0.99	0.000
	CK vs. 400-fold	-69.46	-91.49 to -47.44	***	<0.001	8.730	200-fold vs. 600-fold	0.000	-24.83 to 24.83	ns	>0.99	0.000
	CK vs. 600-fold	-69.46	-91.49 to -47.44	***	<0.001	8.730	400-fold vs. 600-fold	0.000	-24.83 to 24.83	ns	>0.99	0.000

Table S6. Statistical data of matrine field trials

Time	Population reduction rate						Control efficacy					
		Mean Diff.	95.00% CI of diff.	Summary	Adjusted P Value	t		Mean Diff.	95.00% CI of diff.	Summary	Adjusted P Value	t
3d	CK vs. 50-fold	-62.76	-79.84 to -45.69	***	<0.001	10.17	50-fold vs. 125-fold	1.910	-15.49 to 19.31	ns	0.99	0.2775
	CK vs. 125-fold	-61.23	-78.31 to -44.16	***	<0.001	9.924	50-fold vs. 200-fold	8.644	-8.757 to 26.05	ns	0.52	1.256
	CK vs. 200-fold	-54.84	-71.92 to -37.76	***	<0.001	8.888	125-fold vs. 200-fold	6.734	-10.67 to 24.14	ns	0.71	0.9785
5d	CK vs. 50-fold	-57.78	-74.85 to -40.70	***	<0.001	9.365	50-fold vs. 125-fold	0.8985	-16.50 to 18.30	ns	>0.99	0.1306
	CK vs. 125-fold	-57.32	-74.39 to -40.24	***	<0.001	9.290	50-fold vs. 200-fold	12.71	-4.689 to 30.11	ns	0.21	1.847
	CK vs. 200-fold	-46.77	-63.85 to -29.70	***	<0.001	7.581	125-fold vs. 200-fold	11.81	-5.587 to 29.22	ns	0.26	1.717
9d	CK vs. 50-fold	-74.22	-91.29 to -57.14	***	<0.001	12.03	50-fold vs. 125-fold	-3.669	-21.07 to 13.73	ns	0.93	0.5331
	CK vs. 125-fold	-77.37	-94.44 to -60.29	***	<0.001	12.54	50-fold vs. 200-fold	3.085	-14.32 to 20.49	ns	0.96	0.4482

	CK vs. 200-fold	-71.61	-88.68 to -54.53	***	<0.001	11.61	125-fold vs. 200-fold	6.754	-10.65 to 24.16	ns	0.70	0.9813
14d	CK vs. 50-fold	-71.18	-88.26 to -54.11	***	<0.001	11.54	50-fold vs. 125-fold	5.402	-12.00 to 22.80	ns	0.82	0.7849
	CK vs. 125-fold	-67.06	-84.13 to -49.98	***	<0.001	10.87	50-fold vs. 200-fold	1.284	-16.12 to 18.69	ns	>0.99	0.1865
	CK vs. 200-fold	-70.15	-87.23 to -53.08	***	<0.001	11.37	125-fold vs. 200-fold	-4.118	-21.52 to 13.28	ns	0.91	0.5983
21d	CK vs. 50-fold	-69.46	-86.54 to -52.39	***	<0.001	11.26	50-fold vs. 125-fold	0.000	-17.40 to 17.40	ns	>0.99	0.000
	CK vs. 125-fold	-69.46	-86.54 to -52.39	***	<0.001	11.26	50-fold vs. 200-fold	0.000	-17.40 to 17.40	ns	>0.99	0.000
	CK vs. 200-fold	-69.46	-86.54 to -52.39	***	<0.001	11.26	125-fold vs. 200-fold	0.000	-17.40 to 17.40	ns	>0.99	0.000

Table S7. Raw data of the field trial

Azadirachtin	Concentrations	Treatments			Matrine	Concentrations	Treatments		
		1	2	3			1	2	3
Initial population	200	41	53	59	Initial population	50	56	62	50
	400	56	48	74		125	73	91	66
	600	89	57	74		200	76	64	76
3 d	200	33	47	38	3 d	50	11	25	18
	400	44	26	40		125	28	18	28
	600	50	45	36		200	32	32	21
5 d	200	32	35	25	5 d	50	12	20	18
	400	27	22	27		125	25	20	23
	600	36	34	48		200	40	28	20
9 d	200	26	10	8	9 d	50	10	7	1
	400	3	7	7		125	5	8	4
	600	10	14	16		200	3	14	10
14 d	200	6	4	4	14 d	50	1	3	3
	400	4	4	12		125	6	7	6
	600	10	4	5		200	2	5	4
21 d	200	0	0	0	21 d	50	0	0	0
	400	0	0	0		125	0	0	0
	600	0	0	0		200	0	0	0

Beauveria bassiana	Concentrations	Treatments			MbNPV	Concentrations	Treatments		
		1	2	3			1	2	3
Initial population	100	58	56	86	Initial population	400	33	47	49
	500	76	82	66		900	40	80	53
	900	60	42	39		1400	40	38	32
3 d	100	46	46	54	3 d	400	28	35	40
	500	56	63	60		900	32	66	32
	900	43	28	26		1400	30	28	24
5 d	100	30	30	48	5 d	400	14	22	20
	500	46	42	56		900	30	20	15
	900	35	20	28		1400	19	28	19
9 d	100	2	6	3	9 d	400	5	4	3
	500	7	5	6		900	4	2	5
	900	10	5	4		1400	5	4	3
14 d	100	5	4	7	14 d	400	4	5	5
	500	8	3	5		900	6	4	5
	900	3	2	4		1400	4	5	5
21 d	100	0	0	0	21 d	400	0	0	0
	500	0	0	0		900	0	0	0
	900	0	0	0		1400	0	0	0

Bt	Concentrations	Treatments			COMa421	Concentrations	Treatments		
		1	2	3			1	2	3
Initial population	100	43	41	63	Initial population	250	71	73	61
	250	59	38	35		850	56	44	40
	350	49	68	49		1500	69	56	50
3 d	100	36	34	42	3 d	250	52	50	50
	250	37	27	26		850	26	34	25
	350	40	49	40		1500	42	41	44
5 d	100	19	28	32	5 d	250	20	46	50
	250	15	12	17		850	23	18	25
	350	30	35	28		1500	46	46	30
9 d	100	4	3	1	9 d	250	10	10	25
	250	5	0	3		850	10	7	3
	350	6	4	6		1500	1	2	2
14 d	100	0	4	1	14 d	250	6	8	2
	250	10	9	9		850	9	2	4
	350	3	2	1		1500	6	0	2
21 d	100	0	0	0	21 d	250	0	0	0
	250	0	0	0		850	0	0	0
	350	0	0	0		1500	0	0	0
